# Survival strategies of citrus rootstocks subjected to drought

**DOI:** 10.1038/srep38775

**Published:** 2016-12-20

**Authors:** Dayse Drielly Souza Santana-Vieira, Luciano Freschi, Lucas Aragão da Hora Almeida, Diogo Henrique Santos de Moraes, Diana Matos Neves, Liziane Marques dos Santos, Fabiana Zanelato Bertolde, Walter dos Santos Soares Filho, Maurício Antonio Coelho Filho, Abelmon da Silva Gesteira

**Affiliations:** 1 Departamento de Biologia, Centro de Genética and Biologia Molecular, Universidade Estadual de Santa Cruz, Ilhéus, Bahia 45662-900, Brazil; 2 Departamento de Botânica, Instituto de Biociências, Universidade de São Paulo, São Paulo 05508-090, Brazil; 3 Departamento de Ciências Agrárias, Universidade Federal do Recôncavo da Bahia, Cruz das Almas, Bahia 44380-000, Brazil; 4 Embrapa Mandioca e Fruticultura, Cruz das Almas, Bahia, 44380-000, Brazil; 5 Departamento de Ensino, Instituto Federal da Bahia, Campus Eunápolis, Eunápolis, Bahia 45823-431, Brazil

## Abstract

Two citrus rootstocks, Rangpur lime (RL) and Sunki Maravilha mandarin (SM), were analyzed either ungrafted or grafted with their reciprocal graft combinations or with shoot scions of two commercial citrus varieties: Valencia orange (VO) and Tahiti acid lime (TAL). All graft combinations were subjected to distinct watering regimes: well-watered, severe drought and rehydration. Growth and water relation parameters, gas exchange as well as sugar and hormone profiles were determined. Data indicated that RL adopted a dehydration avoidance strategy and maintained growth, whereas SM adopted a dehydration tolerance strategy focused on plant survival. Compared with RL, the leaves and roots of SM exhibited higher concentrations of abscisic acid and salicylic acid, which induced drought tolerance, and accumulation of carbohydrates such as trehalose and raffinose, which are important reactive oxygen species scavengers. SM rootstocks were able to transfer their survival strategy to the grafted shoot scions (RL, VO, TAL). Because of their contrasting survival strategies, RL reached the permanent wilting point more quickly than SM whereas SM recovered from prolonged droughts more efficiently than RL. This is one of the most complete studies of drought tolerance mechanisms in citrus crops and is the first to use reciprocal grafting to clarify scion/rootstock interactions.

Due to long production periods^1^,^2^, citrus crops are subjected to several biotic and abiotic stresses. Drought is one of the most threatening abiotic factors for citrus cultivation, causing decreased plant growth, development and productivity^3^. In addition, drought severity and/or intensity is increasing worldwide^4^.

Rootstock selection and improvement with the goal of increasing plant water use efficiency is an efficient strategy to minimize the effects of climate changes on plant production^5^. Grafting is a millenary technique widely used in several different cultures^6^. A recent study showed the importance of rootstocks for food security by increasing the efficiency of natural (water and soil) resource utilization and decreasing the use of chemical inputs^7^. Rootstocks increase the resistance of citrus crops to biotic and abiotic factors^8^,^9^,^10^. In the case of drought, this resistance is due to physiological changes that lead to changes in leaf water potential, stomatal conductance and hydraulic conductance^8^,^11^,^12^.

Drought-associated dehydration results from an imbalance between root water uptake and water loss via transpiration, leading to decreased gas exchange and, consequently, reduced photosynthetic rates^8^,^13^,^14^,^15^. Perception and signal transduction of abiotic stresses, including drought, is initiated by a signaling cascade termed systemic acquired acclimation (SAA)^15^. SAA results from plant physiological responses that may include changes at the transcriptional, proteomic and post-transcriptional modification levels, as well as metabolic changes and/or metabolite accumulation^16^. These responses to abiotic stresses comprise, for instance, hormonal changes^17^,^18^,^19^,^20^ or the accumulation of solutes such as aminoacids and carbohydrates^16^,^21^,^22^.

Depending on their intrinsic characteristics, plants may adopt different strategies to cope with drought periods. These different strategies can be divided into two major categories, dehydration avoidance and dehydration tolerance, which involve different physiological processes^16^,^23^,^24^. Dehydration avoidance comprises multiple strategies to prevent water loss, such as solute accumulation and cell wall hardening. Dehydration tolerance involves mechanisms to avoid cell damage caused by water loss, such as synthesis of osmoprotectant proteins and solutes, metabolic changes, and detoxification of reactive oxygen species (ROS)^25^,^26^. Whereas dehydration avoidance is focused on the maintenance of plant growth and productivity, dehydration tolerance is focused on plant survival, especially during prolonged drought periods^16^. Rangpur lime (RL) and Sunki Maravilha mandarin (SM) rootstocks exhibit different patterns of soil water uptake and protein profiles under drought conditions^27^,^28^, indicating different drought survival strategies. Therefore, the present study investigated drought survival strategies of scion/rootstock reciprocal combinations between RL and SM and also combination of either RL or SM rootstocks with shoot scions of two commercial citrus varieties: Valencia orange (VO) and Tahiti acid lime (TAL). The influence of scions on rootstocks, and vice versa, was investigated, revealing that rootstocks can transfer their survival strategy to the grafted shoot scions. RL adopted a dehydration avoidance strategy and maintained growth, SM adopted a dehydration tolerance strategy focused on plant survival, which involved critical differences in hormonal and carbohydrate profiles. Due to their contrasting survival strategies, RL reached the permanent wilting point quicker than SM whereas SM recovered from prolonged droughts more efficiently than RL.

## Results

### Morphological and physiological responses of eight different scion/rootstock combinations subjected to drought

Drought was induced by withholding water supply to plants. Plants reached severe stress, as indicated by the leaf water potential (Ψ_L_ ≤ −2.0 MPa), on different days. During the experiment, the air relative humidity decreased slightly; however, no sudden changes in climate conditions were observed (Supplementary Fig. S1). Leaf area values of drought-exposed plants before and after drought application, for all the scion/rootstock combinations tested, indicated no significant interactions between plant combination and water availability conditions, except for comparison group 2 (Supplementary Fig. S2).

**Figure 1:**
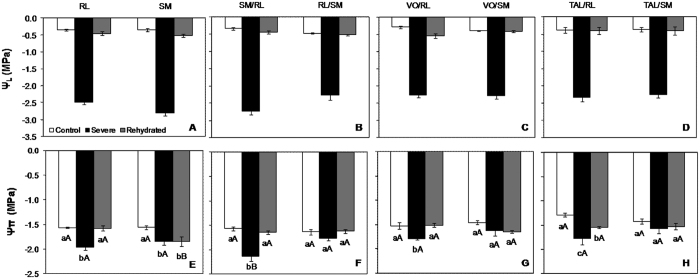
Pre-dawn leaf water potential ((**A**–**D**); Ψ_L_; MPa), and leaf osmotic potential ((**E**–**H**); Ψπ; MPa) of eight scion/rootstock combinations (RL: Rangpur lime; SM: Sunki Maravilha mandarin; VO: Valencia orange; TAL: Tahiti acid lime), under three different water availability conditions: control: Ψ_L_ ≤ 0.5 (white bars); severe drought stress: Ψ_L_ ≥ 2.0 (black bars); and 48-h rehydration (gray bars). Values are averages ± standard errors (n = 3). Different uppercase letters indicate significant differences between combinations, and different lowercase letters indicate significant differences within the same combination, according to the Scott-Knott test (*p* < 0.05).

All plants were harvested at soil Ψ values lower than −1.5 MPa (theoretical wilting point–Supplementary Fig. S3) and at leaf water potential (Ψ_L_) values ≤ −2.0 MPa (Fig. 1A–D), which indicates severe drought stress and was adopted as the threshold Ψ_L_ value for plant harvest in the present study. All control plants, independent of the combination, exhibited Ψ_L_ ≥ −0.5 MPa. Similar values were observed following 48-h rehydration.

**Figure 2:**
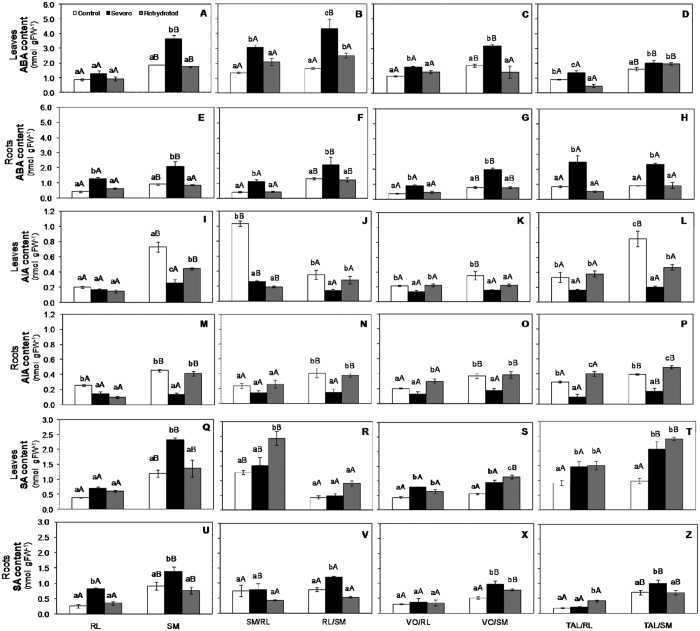
ABA, IAA and SA concentrations in leaves (**A**–**D**,**I**–**L**,**Q**–**T** respectively) and roots (**E**–**H**,**M–P**,**U–Z** respectively) of eight different scion/rootstock combinations under three different water availability conditions: control: Ψ_L_ ≤ 0.5 (white bars); severe drought stress: Ψ_L_ ≥ 2.0 (black bars); and 48-h rehydration (gray bars). RL: Rangpur lime; SM: Sunki Maravilha mandarin; VO: Valencia orange; TAL: Tahiti acid lime. Values are averages ± standard errors (n = 5). Different uppercase letters indicate significant differences between combinations, and different lowercase letters indicate significant differences within the same combination, according to the Scott-Knott test (*p* < 0.05).

A significant interaction between plant combination and drought treatment was observed for leaf osmotic potential (Ψπ) exclusively in comparison groups 1 (Fig. 1E) and 2 (Fig. 1F). Ψπ was significantly lower for SM than for RL following rehydration. RL exhibited significantly different Ψπ values under severe drought, under control conditions, and following rehydration. For SM, the Ψπ values under control conditions were significantly different from the Ψπ values under the remaining water availability conditions. Regarding the reciprocal graft combinations (group 2), SM/RL exhibited significantly lower Ψπ under severe drought than under control conditions and following rehydration, whereas no significant differences were observed for RL/SM. Except for ungrafted SM, no significant differences between different water availability conditions were observed for plants with SM rootstocks (RL/SM, VO/SM and TAL/SM) for any of the tested genotypes. This result indicates that SM rootstocks can determine plant behavior during severe drought stress. All the combinations with RL rootstocks, including ungrafted RL, exhibited significantly lower Ψπ under severe drought than under control conditions or following rehydration. This finding indicates that plants with RL rootstocks exhibited osmotic adjustment compared to plants with SM rootstocks.

Net photosynthetic rate (A), stomatal conductance (Gs), transpiration rate (E) decreased under severe drought compared to control plants or following 24-h rehydration for all tested plant combinations (Table 1). Significant differences in A were observed only for the comparison between RL and SM (Table 1), with SM exhibiting higher A following 24-h rehydration. Significant differences in A between different water availability conditions were observed for all plant combinations tested, except for the comparison between TAL/RL and TAL/SM, which revealed significant differences between severe drought and following rehydration, with A being higher for TAL/SM. It should be noted that no significant differences in A were observed between plants with RL and SM rootstocks under control conditions; however, plants with RL or SM rootstocks exhibited pronounced decreases (78–100%) in A under severe drought compared to control conditions. Following rehydration, A increased 30.2- (RL), 71.6- (SM/RL), 6.9- (VO/RL) and 299.8-fold (TAL/RL) compared to plants under severe drought stress for plants with RL rootstocks, and 4.6- (SM), 6.1- (RL/SM), 4.0- (VO/SM) and 2.6-fold (TAL/SM) for plants with SM rootstocks.

SM exhibited Gs values almost twice as high as RL following 24-h rehydration and no significant differences were observed among the remaining genotypes (Table 1). However, significant differences between different water availability conditions were observed for all plant combinations, except for TAL/RL and TAL/SM, in which Gs was almost twice as high for TAL/SM than for TAL/RL following rehydration. Significant differences in E between different water availability conditions were observed for each plant combination (Table 1). For groups 1 (RL and SM), 2 (SM/RL and RL/SM), and 4 (TAL/RL and TAL/SM), the highest E values were observed under control conditions, followed by rehydration and then severe drought. For group 3 (VO/RL and VO/SM), E under severe drought was significantly different from the control treatment and following 24-h rehydration, but not between the latter two treatments. Two observations regarding transpiration rates should be highlighted. First, plants with RL rootstocks exhibited a stronger recovery of transpiration rates following 24-h rehydration than plants with SM rootstocks, this when is compared the value of E at the severe stress and the increase of this value at after 24-h rehydration. The plants with RL rootstocks exhibited E values 7.5- (RL), 7.3- (SM/RL), 4.1- (VO/RL) and 4.9-fold (TAL/RL) higher than under severe drought stress. Plants with SM rootstocks exhibited E values 2.7- (SM), 3.5- (RL/SM), 3.4- (VO/SM) and 1.9-fold (TAL/SM) higher following rehydration than under severe drought stress. Second, the TAL/SM combinations exhibited the highest transpiration rates during severe drought, which were significantly different from those of TAL/RL. This finding is consistent with the photosynthetic rates and stomatal conductance results for these plant combinations (Table 1).

Significant interactions for intrinsic water use efficiency (A/Gs) were observed for all the comparison groups (Table 1). It should be noted that A and Gs were markedly higher for plants with SM rootstocks than for plants with RL rootstocks under severe drought. No significant differences were observed between plants with RL and SM rootstocks under control conditions or following 24-h rehydration. For instant efficiency of water use (A/E), significant differences were observed between RL and SM only, with higher levels for SM following rehydration and under severe drought. For group 2 (SM/RL and RL/SM), only SM/RL exhibited significant differences in A/E between severe drought stress and under control conditions or following rehydration. For groups 3 (VO/RL and VO/SM) and 4 (TAL/RL and TAL/SM), and also for VO/RL and TAL/RL significant differences between all the tested water availability conditions were observed for, whereas VO/SM and TAL/SM exhibited significant differences between the control treatment and severe drought or following rehydration, but not between the latter two conditions.

### Drought-induced changes in hormone levels

Plant hormones are regulated by environmental changes and by simulated stress conditions, such as drought. Overall, drought, especially severe drought, affected hormone concentrations in all the plant combinations, resulting in decreased or increased concentrations, depending on the hormone (Fig. 2). In addition, hormone concentrations were observed to be higher in leaves than in roots, especially under control conditions, in all plant combinations. Except for the leaf IAA and SA concentrations in SM/RL, plants with SM rootstocks (ungrafted or grafted) exhibited significantly higher or similar concentrations of all tested hormones compared to plants with RL rootstocks (ungrafted or grafted) under control conditions. In addition, rootstocks were observed to influence scion behavior, and vice versa. This finding was particularly evident for the comparisons between the reciprocal graft combinations SM/RL and RL/SM (Fig. 2), confirming the importance of scion/rootstock interactions for plant development and survival in different environments and/or stress conditions.

Both leaf and root ABA concentrations increased under severe drought for all plant combinations (Fig. 2A–H). This result is associated with the decreased stomatal conductance observed under the same conditions. The only exception was RL, which exhibited no significant differences in leaf ABA content between the different water availability conditions. Notably, plants with SM rootstocks (ungrafted or grafted) exhibited higher ABA concentration than plants with RL rootstocks (ungrafted or grafted) under both severe drought stress and control conditions, which may indicate an influence of rootstocks on scion behavior. Furthermore, the influence of scions on rootstock behavior was confirmed by the finding that the reciprocal graft combination SM/RL exhibited pronouncedly higher ABA levels than RL (ungrafted); the difference was not as pronounced for the remaining tested scions grafted onto RL. Similar ABA levels were observed for RL/SM and the ungrafted SM.

Overall, IAA content decreased under severe drought stress compared to control conditions. This finding is associated with the increases observed in ABA concentrations (Fig. 2A–H). It should be noted that under control conditions, plants with SM rootstocks (ungrafted or grafted) exhibited higher leaf and/or root IAA levels than plants with RL rootstocks. However, SM/RL exhibited higher leaf IAA content under control conditions than the reciprocal graft combination RL/SM (Fig. 2J).

SA levels increased under severe drought in all plant combinations, except for RL (Fig. 2Q) and RL/SM (Fig. 2R) in leaves, and SM/RL (Fig. 2V), VO/RL (Fig. 2X) and TAL/RL (Fig. 2Z) in roots. It should be highlighted that the plants with SM rootstocks (ungrafted or grafted) exhibited higher root SA concentrations than those with RL rootstocks (ungrafted or grafted) under severe drought stress (Fig. 2U–Z). Higher leaf SA under severe drought were also observed for SM (Fig. 2Q) and TAL/SM (Fig. 2T) compared to RL (Fig. 2Q) and TAL/RL (Fig. 2T), respectively.

### Drought-induced changes in carbohydrate profile

The leaf and root concentrations of raffinose, trehalose, galactose, fructose, glucose and sucrose for the eight plant combinations and three water availability conditions evaluated are presented in Table 2. Overall, drought resulted in increased carbohydrate concentrations. Raffinose, trehalose and galactose concentrations were higher in roots than in leaves, whereas fructose, glucose and sucrose concentrations were similar in leaves and roots.

Except for RL, SM/RL and TAL/RL, all plant combinations exhibited increased leaf raffinose under severe drought, which was particularly evident in plants with SM rootstocks (Table 2). Except for TAL/SM, all plant combinations with SM rootstocks exhibited significantly higher raffinose content under severe drought; these concentrations decreased to levels similar to the control treatment following 48-h rehydration.

Fluctuations in trehalose levels resembled those observed for raffinose levels. In all plants with SM rootstocks (ungrafted or grafted), leaf and root trehalose levels increased under severe drought and then decreased following 48-h rehydration (Table 2). No significant differences in leaf trehalose content between the three water availability conditions were observed for plants with RL rootstocks (ungrafted or grafted). Root trehalose content under different water availability conditions was significantly higher following 48-h rehydration for SM/RL, and under severe drought stress for VO/RL.

For leaf galactose, significant differences were observed only between RL and SM and between their reciprocal grafts, SM/RL and RL/SM, whereas significant differences in root galactose were observed between VO/RL and VO/SM and between TAL/RL and TAL/SM (Table 2). Sucrose is formed by the combination of fructose and glucose, and overall, the concentrations of these three carbohydrates followed similar tendencies. Interestingly, in plants with SM rootstocks, the leaf concentrations of fructose, glucose and sucrose increased under severe stress and decreased again following 48-h rehydration (Table 2). Root fructose and sucrose levels exhibited a tendency similar to that observed for leaves, with increased concentrations under severe drought stress. However, root glucose increased following 48-h rehydration in plants with SM rootstocks.

## Discussion

In the present work, marked changes in leaf water potential, leaf osmotic potential, gas exchange, hormone and carbohydrate profiles were triggered by drought in distinct scion/rootstock combinations in citrus plants. RL and SM rootstocks were selected based on previous studies indicating their different behaviors when subjected to drought stress^27^,^28^. The reciprocal graft combinations (SM/RL and RL/SM), as well as two commercial scions were used–Valencia orange (VO) and Tahiti acid lime (TAL)–which were grafted onto SM and RL rootstocks, were used to further investigate scion/rootstock interactions. RL rootstocks are widely used by Brazilian citrus producers, and SM and its derivatives may be an alternative for genetic diversification.

### Changes and interactions of gas exchange and hormone profiles and sugars under water deficit

The first drought-induced responses in plants include the stomatal closure (to avoid water loss) and the increased root:shoot ratio^26^, both of which are critically important for plant survival. However, during longer periods of drought, plants lose the ability to balance water uptake and loss, and leaf water potential (Ψ_W_) decreases. Two strategies may then be adopted: (i) dehydration avoidance or (ii) dehydration tolerance. Dehydration avoidance is characterized by solute accumulation and cell wall hardening to decrease water loss. Dehydration tolerance involves the production of protective solutes and proteins, metabolic changes and ROS detoxification to avoid damages caused by cellular water loss^16^. In the present study, the tested rootstocks, RL and SM, were observed to exhibit different strategies to tolerate/survive drought.

Both tested rootstocks, RL and SM, and their different graft combinations exhibited decreased leaf water potential (Ψ_W;_ Fig. 1A–D) and soil matric potential (Supplementary Fig. S3A–D) after water withholding, indicating that they were affected by the severe drought treatment. In addition, A, Gs and E were observed to decrease under severe drought in all plant combinations (Table 1). Similar findings have been previously reported for different citrus genotypes under drought conditions^11^,^26^. No significant differences in A, Gs or E were observed between RL and SM rootstocks (grafted or ungrafted) under control conditions, indicating that RL and SM possess similar capacities for CO_2_ absorption and control of water loss by transpiration under high soil water availability conditions. Significant differences were observed between the ungrafted rootstocks (RL and SM) following 24-h rehydration; the plants with the rootstocks formed by SM exhibited higher A and Gs, indicating that SM has a greater capacity to recover from severe drought. In addition, plants with SM rootstocks exhibited higher A/ Gs and A/E levels under severe drought than plants with RL rootstocks, indicating that even under field conditions, where RL was observed to maintain the production (data not shown), SM decreases production levels and uses the available water more efficiently to maintain metabolic processes and survive prolonged drought periods. A/Gs and A/E are very important for plant development under water limited conditions, such as in semi-arid regions. Studies have identified citrus plants with high A/Gs, such as those exhibited by SM, as suitable for semi-arid regions and/or regions with partial irrigation methods^8^,^29^,^30^,^31^.

ABA is considered the primary hormone involved in regulation of plant responses to abiotic stresses, especially drought^32^. ABA levels were observed to increase in both roots (all tested plant combinations) and leaves (except for RL) (Fig. 2A–H). One of the main functions of ABA is the induction of stomatal closure, which occurs through the inhibition of the flow of potassium ions into guard cells to control water loss due to transpiration^33^,^34^. This phenomenon also results in decreased photosynthetic rates. An interaction between increased leaf and root ABA contents and decreased gas exchange was observed under severe drought stress for all combinations tested.

Except for the reciprocal grafts SM/RL and RL/SM, plants with SM rootstocks exhibited significantly higher ABA content than plants with RL rootstocks under control conditions. It was long believed that endogenous ABA under high water availability could decrease shoot growth. However, normal levels of endogenous ABA have been reported to be required to maintain shoot growth^35^. No significant differences in Gs, A or E were observed under control conditions between plants with SM rootstocks, which exhibited higher ABA levels, and plants with RL rootstocks. In addition, in TAL/RL, the TAL scions, which are tall and exhibit vigorous growth and abundant foliage, induced the RL rootstocks to produce ABA levels under control and severe drought conditions (Fig. 2H) that were at least twice the levels of the remaining combinations with RL rootstocks (RL, SM/RL and VO/RL; Fig. 2E–G). This finding indicates that due to their characteristics, TAL scions trigger higher production of ABA by RL rootstocks in an attempt to control water loss by TAL shoots.

Interestingly, under severe drought, all plants with SM rootstocks (grafted and ungrafted) (Fig. 2A–D) exhibited higher leaf ABA than plants with RL rootstocks (grafted or ungrafted). Under severe drought, SM scions grafted onto RL exhibited leaf ABA levels approximately 2.5-fold higher than ungrafted RL. ABA has been frequently reported to be produced in roots and to translocate into leaves, especially under drought conditions^20^,^27^. However, other studies have shown that ABA can also be produced in shoots and/or guard cells, protecting plants from water loss by inducing stomatal closure^36^,^37^,^38^,^39^,^40^. Therefore, these data indicate that ABA production can be stimulated in SM leaves under severe drought conditions, which, in turn, leads to increased ABA production in grafted scions as well.

Auxins, particularly IAA, play critical roles in numerous plant growth and development responses^41^. IAA is mainly produced in young shoot regions and is redistributed via both cell-to-cell polar transport and non-polar transport in phloem to other plants regions, such as the roots, whose growth and development is strongly modulated by this hormone^42^. Except for RL/SM, leaf IAA levels under control conditions were higher for plants with SM rootstocks than with RL rootstocks (Fig. 2I–L). SM/RL exhibited higher IAA content than the reciprocal combination under control conditions, with shoots exhibiting a prevalence of SM characteristics (ungrafted), whereas the root IAA concentration was more similar to those observed for ungrafted RL. The decreased IAA levels observed under severe drought may result from the effect of water deficit on IAA polar transportation^41^. Studies with different crops have shown increased ABA and decreased IAA levels under osmotic stress due to drought or salinity stress^43^,^44^. Here, increased ABA and decreased IAA concentrations were observed under severe drought for all plant combinations. Since IAA affects stomatal aperture^34^, the reductions in IAA content observed in drought-exposed plants may facilitate the promotive action of ABA on stomatal closure and, consequently, on the minimization of water loss due to transpiration.

SA has generally been reported to be critical for tolerance to biotic stresses, usually being produced at sites of infection^45^,^46^. However, SA has also been observed to play significant roles in tolerance to abiotic stresses^47^, plant growth and development, and stomatal movements^48^,^49^. Recent studies based on exogenous applications of SA^47^,^50^ or using SA-deficient or -overproducer mutants^51^,^52^ have also shown that SA increases drought tolerance in plants. This increased drought tolerance is directly related to the induction of ROS production mediated by peroxidases and the promotion of stomatal closure^52^. Stomatal closure induced by SA is also related to defense against pathogens by preventing pathogen entry through the stomata^53^. Here, several scion/rootstock combinations exhibited increased SA concentrations during severe drought (Fig. 2). Therefore, these increased levels of SA in drought-exposed citrus plants may also have facilitated stomatal closure under these environmental circumstances (Table 1), very likely acting in conjunction with the increased ABA levels also detected under drought conditions (Fig. 2A–H). Since all plant combinations that did not exhibit increased SA under severe drought had RL rootstocks and/or shoots, SA might be involved in the different physiological strategies adopted by RL and SM genotypes under water-limited conditions. Under drought, RL adopts a strategy of maintenance of plant growth/production, whereas SM adopts a strategy focused on plant survival, i.e., ensuring that plants survive the stress period.

Interestingly, SA supplementation before stress was observed to increase ABA contents^54^, and both hormones are known to induce ROS production and stomatal closure. An SA increase can directly result in higher drought tolerance for both analyzed genotypes and for the different plant combinations. However, the increase in SA was more pronounced in plants with SM rootstocks or scions (SM/RL) than in those with RL rootstocks. Induction of SA accumulation may play a protective role during water stress^49^. SM may therefore possess a more efficient system to protect against damages caused by drought than RL, i.e., a protective mechanism to ensure plant survival under drought conditions.

Most plant combinations exhibited increased carbohydrate concentrations under severe drought stress in both leaves and roots (Table 2). Carbohydrates, especially sucrose, the main product of photosynthesis, play a central role in plant life by affecting plant development, growth, storage, signaling, and acclimatization to biotic and abiotic stresses^55^. In general, drought increases the concentrations of soluble carbohydrates, which are synthesized in response to osmotic stress and act as osmoprotectants by stabilizing cell membranes and maintaining plant turgor^56^. Some of the main functions of carbohydrates, such as sucrose, raffinose and trehalose, involve replacing the water loss, binding to the polar ends of membrane phospholipids and maintaining cell turgor. In addition, carbohydrate accumulation during osmotic stress affects ROS production. This relation between carbohydrates and ROS may be mediated by both increases and decreases in carbohydrate cell contents^57^. Since excessive ROS causes oxidative damage to cells^58^, the production of ROS scavengers is of great importance.

Soluble carbohydrates produced during stress were previously thought to be direct or indirect signals for the production of ROS scavengers and/or repair enzymes. However, recent studies have suggested that soluble carbohydrates, namely raffinose and trehalose, may act as true ROS scavengers^59^,^60^. In addition to being osmoprotectants, the raffinose family of oligosaccharides (RFOs), has been recently described to have antioxidant action by acting as ROS scavengers and inducing higher tolerance in plants under osmotic stress^61^,^62^. Raffinose levels were observed to increase in leaves and roots under severe drought stress, especially in plants with SM rootstocks, with more pronounced increases in root tissues (Table 1). Roots are the first plant organs to perceive water stress, initiating a series of reactions that help plants prevent or tolerate stress. The pronounced increase in raffinose in leaves of drought-exposed citrus plants may be associated with its roles both in signaling and as ROS scavengers.

Trehalose can be maintained at high levels without damaging cell metabolism because of its high solubility and non-reducing nature^60^. In addition, recent studies using *Arabidopsis thaliana* mutants have shown that trehalose metabolism plays an important role in the response of guard cells to ABA by controlling stomatal conductance^60^,^63^,^64^. This finding is in agreement with the present study, in which high levels of ABA (Fig. 2A–H) and trehalose (Table 2) were observed to coincide with low stomatal conductance (Table 1). This is the first report of increased trehalose and raffinose concentrations in citrus plants during drought. This finding is of great importance for the understanding of drought stress tolerance in citrus plants, and suggests that further, more specific, studies of these carbohydrates would be valuable.

### The evaluated citrus plants have different survival strategies to drought

Neves *et al*. 2013^27^ and Oliveira *et al*. 2015^28^ measured physiological parameters, ABA concentrations, gene expression of proteins associated with the ABA biosynthetic pathway, and protein profiles of RL and SM rootstocks grown in pots under drought conditions, and they observed that RL and SM exhibited different responses to drought stress. SM, compared with RL, exhibited higher ABA concentrations and greater numbers of differentially expressed proteins in response to drought. In addition, proteins found exclusively in SM were involved with DNA repair and processing^28^, whereas RL exhibited up-regulation of proteins responsible for transportation, protein metabolism, stress response and proteolysis. These studies are in agreement with the present results. Overall, when compared plants with RL rootstocks and plants with SM rootstocks, this latter exhibited high levels of hormones important for the induction of drought tolerance, such as ABA and SA (Fig. 2A–B,E–F), and high levels of carbohydrates, such as sucrose, raffinose and trehalose, that help prevent the cell damage caused by drought stress and maintain cell metabolism and turgor (Tab. 2). Since decreased water availability under field conditions is intensified by evaporation, when these decreases are systematic and continuous, even genotypes such as SM, which possess drought survival mechanisms based on water saving, will also suffer from drought due to evaporation. It is therefore believed that under severe and long periods of drought, RL will reach the permanent wilting point sooner than SM because the drought survival mechanism of SM is more effective under prolonged drought stress.

**Figure 3:**
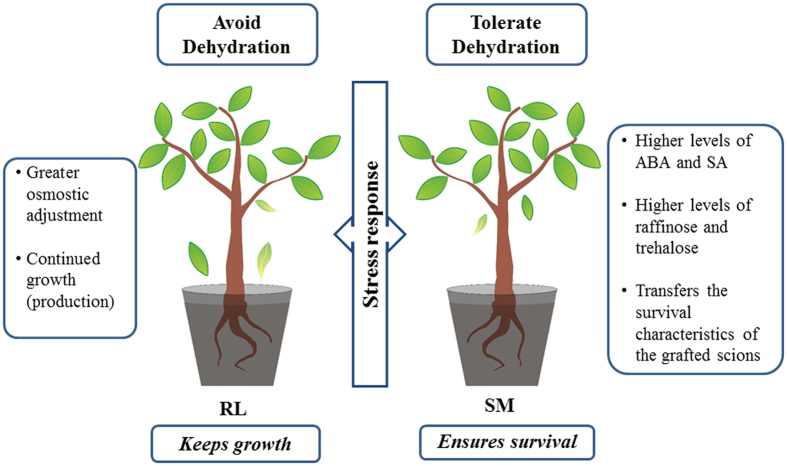
Strategies of RL and SM rootstocks during drought. The physiological responses of drought avoidance (RL) and drought tolerance (SM) are described in the lateral boxes.

Our data also indicates that SM has stronger protective mechanisms than RL, which may confer an advantage to this rootstock under conditions of prolonged drought (Fig. 3). In addition, the lower hormone and carbohydrate concentrations observed for RL than for SM may facilitate plant growth in RL and, therefore, optimizes plant production in this genotype. This finding was confirmed in the field (data not shown), indicating that RL adopts a strategy of dehydration avoidance. Because SM has particularly robust mechanisms of stress protection, it develops a strategy of dehydration tolerance, which may decrease plant production. The selection of rootstocks that guarantee plant survival and maintain plant growth and production rates under prolonged drought conditions is essential for sustainable citrus production; therefore, hybridization between genotypes with these characteristics may be the key to obtaining these varieties.

The present study is, to our knowledge, the first study conducted in citrus plants using reciprocal grafting to clarify scion/rootstock interactions and is one of the most complete studies of drought tolerance in citrus plants performed to date. Among other key findings, our data show that all rootstocks tested, especially SM, tend to modify scion behavior (VO, TAL, and reciprocal grafts) into behavior similar to that exhibited by the ungrafted rootstock.

## Methods

### Plant material and drought treatment

Two rootstocks, *Citrus limonia* Osb. (Rangpur lime) and *Citrus sunki* (Sunki Maravilha mandarin), which exhibit different responses to drought^27^,^28^, and two commercial scions, *Citrus sinensis (L.)* Osb. (Valencia orange) and *Citrus latifolia* Tanaka (Tahiti acid lime), which are economically important worldwide and locally, respectively, were used. Rootstocks were obtained by seed germination, and scions were obtained from buds of healthy mother plants from the Citrus Germplasm Bank (Banco de Germoplasma de Citros) of Embrapa Cassava and Fruit Crops (Embrapa Mandioca e Fruticultura). Bud grafting was performed when the rootstocks were approximately 6 months old. Eight different combinations were tested and they are presented in the Table 3. Following grafting, plants were transferred into 45-L pots containing Plantmax^®^, washed sand and clay (2:1:1). The plants were maintained under an anti-aphid screen with daily irrigation. NPK and micronutrient fertilizers were applied every two weeks until the plants were 2 years old.

Following this period, plants were homogenized by pruning the scions. After one month, the plants were divided into two groups: (i) control treatment: plants grown in soil kept at field capacity with constant irrigation and (ii) drought treatment: plants grown without irrigation. The pots were covered with transparent plastic and aluminum foil to avoid water loss due to evaporation. The experiment lasted 17 days, with drought developing gradually as the soil water content decreased. Soil moisture was monitored daily using a time domain reflectometry (TDR) probe. When the leaf water potential of the plants became lower than −2.0 MPa, they were harvested, and rehydration of these plants was started. The plants reached water deficit on different days. Plants subjected to rehydration were harvested 48-h after rewatering.

### Leaf water potential, leaf osmotic potential and matric potential

Leaf water potential (Ψ_L_) was determined before dawn using a Scholander pressure chamber (m670, PMS Instrument Co., Albany, OR, USA). Ψ_L_ was measured every other day after confirming that photosynthetic parameters were decreasing via measurements using an infrared gas analyzer (IRGA). Leaves were detached from the plants using a stylus and were immediately used for Ψ_L_ measurements. Leaves and roots of plants under severe drought were harvested when they reached Ψ_L_ ≤ −2.0 MPa. Ψ_L_ was also measured for control plants and following 48-h rehydration, after which roots and leaves were harvested.

Leaf osmotic potential (Ψπ) was measured using a Vapro 5520 vapor pressure osmometer (Wescor, Inc., Logan, USA) calibrated using NaCl standards with known concentrations (mmol kg^−1^). Fresh leaves were collected from the middle third of the plants and were macerated, pressed, and filtered to extract the sap. A 10-μL aliquot was used to determine tissue osmolarity. Ψπ values were obtained in mmol kg^−1^ and converted into osmotic potential using the Van’t Hoff equation:


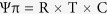


where R is the ideal gas constant (0.00813), T is temperature (K), and C is the leaf extract concentration (mmol kg^−1^, converted to moles).

Soil matric potential (soil Ψ) was determined in function of the soil water content moisture values (measured daily with a TDR probe) based on soil water release curve (determined by pressure plate extractors and WP4 Dewpoint PotentiaMeter dew-point mirror psychrometer.Decagon Devices Inc.)

### Leaf area

Leaf area of drought-exposed plants was measured at the beginning and end (following stress application) of the experiment. The total leaf area was obtained by measuring the length and width once every five leaf of each plant multiplying with a correction factor of 0.72^27^. The total leaf area was determined by summing all the leaf areas obtained for each plant and multiplying the total by five. The total leaf area was obtained in cm^2^ and converted into m^2^.

### Photosynthetic parameters

Net photosynthetic rate (A), stomatal conductance (Gs) and transpiration (E) were measured every other day in fully expanded mature leaves that had previously been selected and marked. Gas exchange measurements (A, Gs and E) were performed using an LCpro-SD portable IRGA (ADC BioScientific Limited, UK) at 1000 μmol photons m^−2^ s^−1^ photosynthetically active radiation (PAR) and ambient leaf temperature, air humidity and CO_2_ concentration. Measurements were performed between 8 AM and 11 AM in two leaves of each plant once the readings had stabilized. Gas exchange measurements in rehydrated plants were performed following 24-h rehydration.

### Hormonal measurements

Endogenous indoleacetic acid (IAA), salicylic acid (SA) and abscisic acid (ABA) levels were determined by gas chromatography tandem mass spectrometry-selected ion monitoring (GC-MS-SIM). Leaf and root samples (50–100 mg FW) were extracted as described in ref. 65. Approximately 0.25 μg of the labeled standards [^2^H_6_]ABA (OlChemIm Ltd.), [^13^C_6_]IAA (Cambridge Isotopes, Inc.) and [^2^H_6_]SA (Cambridge Isotopes, Inc.) was added to each sample as internal standards. For ABA quantification, aliquots of the extract were methylated as described in ref. 65. For IAA and SA quantifications, aliquots were evaporated and resuspended in 50 μL of pyridine, followed by a 60-min derivatization at 92 °C using 50 μL of *N*-*tert*-butyldimethylsilyl-*N*-methyltrifluoroacetamide (with 1% *tert*-butyldimethylchlorosilane). Analysis was performed on a gas chromatograph as in ref. 66. Ions with a mass ratio/charge (m/z) of 244, 202 and 130 (corresponding to endogenous IAA); 250, 208 and 136 (corresponding to [^13^C_6_]-IAA); 309, 195 and 209 (corresponding to endogenous SA); 315, 201 and 215 (corresponding to [^2^H_6_]SA); 190, 162 and 134 (corresponding to endogenous ABA); and 194, 166 and 138 (corresponding to [^2^H_6_]ABA) were monitored.

### Sugar profiling

For soluble carbohydrate profiling, leaf and root samples (50–100 mg FW) were extracted and analyzed as described in ref. 67.

### Statistical analysis

A completely randomized experimental design (CRD) was used, with 3 replicates for the control group and 3 replicates for the groups subjected to drought (for each of the eight tested combinations), for a total of 48 plants. The eight combinations were divided into four comparison groups, each with two combinations, according to the similarity between them. Comparison group 1 consisted of the two ungrafted rootstocks (RL and SM), group 2 was the reciprocal grafts of the two rootstocks (SM/RL and RL/SM), group 3 was Valencia orange scions grafted onto the two rootstocks (VO/RL and VO/SM), and group 4 was Tahiti acid lime scions grafted onto the two rootstocks (TAL/RL and TAL/SM). Analysis of variance (ANOVA) followed by the Scott-Knott test was performed for each comparison group to test for significant differences between combinations within each comparison group, significant differences between different water availability treatments for each combination, and significant interactions between plant combination and drought treatment, with significance defined as *p* ≤ 0.05. Six replicates were performed for the photosynthetic parameters (n = 6), three each for leaf water potential, leaf osmotic potential, soil matric potential, and leaf area (n = 3) and five for hormone contents and carbohydrate profiles (n = 5).

## Additional Information

**How to cite this article**: Santana-Vieira, D. D. S. *et al*. Survival strategies of citrus rootstocks subjected to drought. *Sci. Rep.* **6**, 38775; doi: 10.1038/srep38775 (2016).

**Publisher's note:** Springer Nature remains neutral with regard to jurisdictional claims in published maps and institutional affiliations.

## Supplementary Material

Supplementary Information

## Figures and Tables

**Table 1:** Physiological parameters of eight different scion/rootstock combinations under three different water availability conditions: Control: Ψ_L_ ≤ 0.5; severe drought stress: Ψ_L_ ≥ 2.0; and 48-h rehydration.

Comparison group	Combinations	Condictions	A μmol m^−2^s^−1^	Gs μmol m^−2^s^−1^	E μmol m^−2^s^−1^	A/Gs	A/E
1	RL	C	8.28 ± 0.61cA	0.17 ± 0.01cA	2.16 ± 0.16cA	48.45 ± 1.36bA	3.84 ± 0.05cA
		S	0.12 ± 0.13aA	0.00 ± 0.00aA	0.18 ± 0.09aA	13.83 ± 8.91aA	0.93 ± 0.37aA
		R	3.57 ± 0.51bA	0.04 ± 0.01bA	1.35 ± 0.17bA	82.19 ± 2.27cA	2.65 ± 0.30bA
	SM	C	7.71 ± 0.58cA	0.15 ± 0.02cA	1.98 ± 0.12cA	51.97 ± 2.70aA	3.90 ± 0.22bA
		S	1.22 ± 0.18aA	0.01 ± 0.00aA	0.52 ± 0.06aA	99.25 ± 14.04bB	2.47 ± 0.34aB
		R	5.59 ± 0.19bB	0.08 ± 0.00bB	1.42 ± 0.06bA	72.00 ± 2.70aA	3.94 ± 0.11bB
2	SM/RL	C	5.38 ± 0.98cA	0.12 ± 0.02cA	1.69 ± 0.19cA	43.38 ± 2.56bA	3.08 ± 0.31aA
		S	0.05 ± 0.18aA	0.00 ± 0.00aA	0.19 ± 0.02aA	0.00 ± 0.00aA	1.29 ± 0.48bA
		R	3.46 ± 0.31bA	0.04 ± 0.00bA	1.39 ± 0.11bA	84.66 ± 10.63cA	2.57 ± 0.29aA
	RL/SM	C	6.60 ± 0.41cA	0.16 ± 0.01cB	1.97 ± 0.06cA	41.69 ± 2.65aA	3.36 ± 0.19aA
		S	0.66 ± 0.27aA	0.01 ± 0.00aA	0.42 ± 0.08aA	63.03 ± 11.20bB	2.18 ± 0.62aA
		R	4.03 ± 0.68bA	0.06 ± 0.01bA	1.49 ± 0.28bA	66.06 ± 5.34bA	2.89 ± 0.37aA
3	VO/RL	C	5.17 ± 0.38cA	0.11 ± 0.01cA	1.60 ± 0.10bA	46.38 ± 2.00aA	3.24 ± 0.14cA
		S	0.54 ± 0.20aA	0.01 ± 0.01aA	0.39 ± 0.12aA	31.97 ± 7.95aA	1.10 ± 0.28aA
		R	3.74 ± 0.54bA	0.05 ± 0.00bA	1.60 ± 0.24bA	70.24 ± 8.71aA	2.37 ± 0.11bA
	VO/SM	C	5.62 ± 0.33cA	0.12 ± 0.01cA	1.67 ± 0.08bA	48.44 ± 2.36aA	3.40 ± 0.23bA
		S	1.07 ± 0.23aA	0.01 ± 0.00aA	0.48 ± 0.03aA	107.33 ± 23.37bB	2.21 ± 0.46aA
		R	4.26 ± 0.71bA	0.06 ± 0.01bA	1.62 ± 0.26bA	75.56 ± 6.34bA	2.62 ± 0.18aA
4	TAL/RL	C	7.34 ± 0.30cA	0.17 ± 0.01cA	2.02 ± 0.08cA	44.81 ± 2.43aA	3.65 ± 0.13cA
		S	0.01 ± 0.11aA	0.00 ± 0.00aA	0.25 ± 0.05aA	9.64 ± 4.00aA	0.66 ± 0.43aA
		R	2.50 ± 0.30bA	0.03 ± 0.00bA	1.22±0.02bA	84.35 ± 6.89bA	2.06 ± 0.25bA
	TAL/SM	C	7.69 ± 0.20cA	0.17 ± 0.01cA	2.11 ± 0.17cA	47.30 ± 3.04aA	3.75 ± 0.27bA
		S	1.67 ± 0.30aB	0.02 ± 0.00aA	0.79 ± 0.13aB	102.24 ± 35.55aB	2.23 ± 0.42aB
		R	4.32 ± 0.56bB	0.06 ± 0.01bB	1.54 ± 0.17bA	69.62 ± 4.36aA	2.79 ± 0.13aA

Treatments were divided into four comparison groups. A: photosynthetic rate (μmol m^2^ s^−1^); Gs: stomatal conductance (mol m^2^ s^−1^); E: transpiration rate (mmol m^2^ s^−1^); A/Gs: intrinsic water use efficiency; A/E: water use efficiency. RL: Rangpur lime; SM: Sunki Maravilha mandarin; VO: Valencia orange; TAL: Tahiti acid lime. Values are averages ± standard errors (n = 6). Different uppercase letters indicate significant differences between combinations, and different lowercase letters indicate significant differences within the same combination, according to the Scott-Knott test (*p* < 0.05).

**Table 2:** Raffinose, trehalose, galactose, fructose, glucose and sucrose concentrations in leaves and roots of eight different scion/rootstock combinations under three different water availability conditions.

Comparison group	Combinations	Tissue	Condictions	Raffinose μg gFW^−1^	Trehalose μg gFW^−1^	Galactose μg gFW^−1^	Fructose μg gFW^−1^	Glucose μg gFW^−1^	Sucrose μg gFW^−1^
1	RL	Leaves	C	4.44 ± 1.32aA	8.01 ± 0.68aA	4.07 ± 1.08aA	830.54 ± 39.81bA	351.47 ± 23.42bA	4448.87 ± 363.14bA
		Leaves	S	5.82 ± 0.86aA	5.89 ± 1.54aA	5.43 ± 1.49aA	521.82 ± 28.96aA	285.99 ± 10.37aA	4726.54 ± 366.70bA
		Leaves	R	6.15 ± 1.29aA	4.47 ± 0.89aA	7.68 ± 1.03aB	510.48 ± 49.25aA	229.85 ± 15.55aA	3430.37 ± 259.87aA
		Roots	C	10.87 ± 2.33aA	12.01 ± 3.27aA	10.91 ± 2.76aA	853.87 ± 38.13aA	315.51 ± 11.02aA	4981.34 ± 323.75aA
		Roots	S	15.26 ± 2.26aA	8.12 ± 2.51aA	18.20 ± 1.78aA	1255.91 ± 43.78bA	415.22 ± 15.62bA	7269.38 ± 159.31bA
		Roots	R	16.61 ± 2.47aA	11.30 ± 2.01aA	14.41 ± 2.73aA	919.30 ± 97.70aA	471.78 ± 20.60bA	3789.03 ± 260.05aA
	SM	Leaves	C	10.17 ± 2.05aB	9.53 ± 0.55bA	10.38 ± 0.62bB	810.47 ± 32.10bA	308.84 ± 12.00aA	6170.78 ± 530.66bB
		Leaves	S	14.96 ± 1.12bB	22.22 ± 1.57cB	9.28 ± 1.43bB	979.21 ± 36.32cB	560.37 ± 48.01bB	8727.67 ± 215.98cB
		Leaves	R	6.81 ± 1.41aA	5.42 ± 1.71aA	4.02 ± 1.00aA	617.22 ± 30.71aA	372.27 ± 16.30aB	5074.45 ± 344.57aB
		Roots	C	11.87 ± 2.08aA	21.98 ± 2.68bB	12.96 ± 1.74aA	1233.95 ± 62.42aB	295.84 ± 28.74aA	8206.05 ± 1365.32bB
		Roots	S	37.28 ± 3.08bB	45.15 ± 2.29aB	16.20 ± 3.07aA	1595.06 ± 61.04bB	442.67 ± 11.16bA	9313.46 ± 109.04bB
		Roots	R	11.62 ± 0.72aA	22.88 ± 2.14bB	17.84 ± 2.79aA	1096.71 ± 12.53aB	753.19 ± 26.34cB	3907.82 ± 95.05aA
2	SM/RL	Leaves	C	4.52 ± 1.33aA	5.23 ± 1.32aA	2.97 ± 0.43aA	583.86 ± 24.49bA	323.32 ± 15.27aA	4107.64 ± 238.41aA
		Leaves	S	8.60 ± 1.95aA	6.38 ± 1.82aA	6.80 ± 1.43bA	818.06 ± 40.31cA	546.01 ± 16.41bB	9920.11 ± 311.11bA
		Leaves	R	4.46 ± 0.62aA	8.08 ± 1.20aB	6.07 ± 0.92bA	408.74 ± 14.10aA	320.48 ± 12.30aA	3477.40 ± 242.05aA
		Roots	C	11.45 ± 2.39aA	25.30 ± 1.14bA	15.43 ± 1.96bA	1153.57 ± 52.96aA	446.95 ± 36.54bA	4546.77 ± 151.59aA
		Roots	S	20.40 ± 3.76aA	23.17 ± 1.39bA	17.14 ± 2.25bA	1561.78 ± 150.17bA	366.93 ± 9.57aA	7974.53 ± 372.71bA
		Roots	R	16.40 ± 1.58aA	7.77 ± 1.67aA	7.83 ± 1.65aA	1080.29 ± 101.94aA	367.89 ± 25.41aA	4575.63 ± 824.16aB
	RL/SM	Leaves	C	6.09 ± 1.46aA	3.37 ± 0.93aA	9.03 ± 1.26bB	623.47 ± 22.62aA	268.34 ± 3.19aA	4408.49 ± 216.20aA
		Leaves	S	23.49 ± 2.58cB	14.95 ± 1.18bB	6.59 ± 1.15aA	1036.62 ± 64.01bB	438.93 ± 42.94cA	8297.20 ± 1449.94bA
		Leaves	R	13.31 ± 1.16bB	2.04 ± 1.03aA	5.39 ± 0.28aA	589.87 ± 13.52aB	343.02 ± 11.65bA	4415.87 ± 81.27aA
		Roots	C	17.11 ± 1.41aA	24.54 ± 3.08bA	16.13 ± 1.30aA	1016.60 ± 42.87aA	377.65 ± 25.31aA	5999.95 ± 301.23bB
		Roots	S	37.99 ± 6.63bB	42.22 ± 2.55cB	12.81 ± 2.28aA	1756.84 ± 89.25bA	501.51 ± 22.33bB	8793.19 ± 464.69cA
		Roots	R	17.08 ± 1.46aA	14.75 ± 1.36aB	11.28 ± 2.06aA	1002.54 ± 44.02aA	609.30 ± 11.63cB	3134.63 ± 85.73aA
3	VO/RL	Leaves	C	5.96 ± 1.20aA	6.24 ± 0.89aA	4.27 ± 0.72aA	596.26 ± 12.71bB	314.67 ± 11.74bB	4302.79 ± 89.72bA
		Leaves	S	13.94 ± 0.63bA	8.52 ± 0.61aA	7.16 ± 0.74aA	634.80 ± 42.11bA	465.74 ± 12.79cB	5600.13 ± 97.60cA
		Leaves	R	6.49 ± 1.36aA	5.87 ± 1.60aA	5.41 ± 1.19aA	499.20±22.16aA	232.35 ± 10.83aA	3061.09±132.70aA
		Roots	C	17.01 ± 2.09bA	11.53 ± 2.95aA	17.53 ± 1.22bB	1090.50 ± 95.09bA	264.88 ± 19.93aA	2843.78 ± 89.17aA
		Roots	S	7.59 ± 3.00aA	26.24 ± 1.92bA	16.92 ± 1.42bA	1288.02 ± 80.42bA	448.73 ± 30.21bA	5349.44 ± 327.58bA
		Roots	R	9.35 ± 2.71aA	6.91 ± 2.16aA	11.24 ± 1.50aA	789.15 ± 76.33aA	633.31 ± 28.39cA	3064.98 ± 230.06aA
	VO/SM	Leaves	C	3.59 ± 1.42aA	8.92 ± 0.63aA	4.14 ± 0.63aA	513.25 ± 15.54aA	266.61 ± 2.38aA	4197.96 ± 245.72aA
		Leaves	S	11.87 ± 2.26bA	20.15 ± 0.96bB	9.21 ± 1.66bA	933.35 ± 20.65cB	408.87 ± 24.95bA	8194.20 ± 424.44cB
		Leaves	R	13.91 ± 2.76bB	6.76 ± 1.09aA	3.31 ± 0.34aA	653.83 ± 19.30bB	247.98 ± 16.40aA	5485.43 ± 213.77bB
		Roots	C	12.10 ± 2.08aA	19.94 ± 2.13aB	10.31 ± 1.70aA	1075.96 ± 66.40aA	419.88 ± 27.83aB	4959.96 ± 514.00bB
		Roots	S	29.36 ± 2.61bB	56.52 ± 3.68bB	13.49 ± 2.91aA	1794.25 ± 128.95bB	442.66 ± 33.97aA	9111.56 ± 316.84cB
		Roots	R	14.00 ± 2.02aA	20.82 ± 1.15aB	15.11 ± 3.22aA	959.60 ± 81.14aA	886.51 ± 105.94bB	3773.33 ± 137.59aA
4	TAL/RL	Leaves	C	4.89 ± 1.21aA	4.42 ± 1.02aA	5.53 ± 1.52aA	643.16 ± 12.98bB	295.67 ± 4.86bB	4369.74 ± 181.29aA
		Leaves	S	8.29 ± 2.04aA	6.47 ± 1.55aA	4.42 ± 1.60aA	659.18 ± 57.47bA	435.72 ± 8.84cA	7040.65 ± 531.00bA
		Leaves	R	10.56 ± 0.28aA	3.96 ± 1.56aA	6.80 ± 1.10aA	491.86 ± 16.66aA	214.50 ± 8.52aA	3844.72 ± 874.24aA
		Roots	C	15.40 ± 3.41aA	10.85 ± 3.22aA	15.51 ± 1.46bA	1240.62 ± 58.60bA	349.73 ± 15.41aA	3507.84 ± 489.62aA
		Roots	S	12.94 ± 2.73aA	11.29 ± 2.34aA	15.58 ± 3.15bA	1230.18 ± 58.02bA	425.08 ± 15.06aA	5968.55 ± 236.56bA
		Roots	R	13.01 ± 0.71aA	14.96 ± 0.60aA	6.12 ± 1.45aA	673.54 ± 17.41aA	367.15 ± 36.49aA	3396.58 ± 161.08aA
	TAL/SM	Leaves	C	1.64 ± 0.74aA	5.67 ± 1.73aA	9.36 ± 1.10bB	537.87 ± 8.56aA	227.22 ± 13.48aA	4013.80 ± 173.12aA
		Leaves	S	21.09 ± 1.74bB	15.79 ± 1.51bB	5.24 ± 0.66aA	903.15 ± 16.04cB	421.91 ± 18.60cA	8008.57 ± 100.37cA
		Leaves	R	20.73 ± 3.14bB	6.44 ± 0.62aA	6.19 ± 1.00aA	636.78 ± 5.12bB	266.83 ± 13.28bB	5326.29 ± 162.63bB
		Roots	C	14.09 ± 2.55aA	29.53 ± 3.62bB	10.16 ± 2.05aA	1154.45 ± 94.46aA	327.59 ± 13.72aA	5266.93 ± 181.18bB
		Roots	S	20.29 ± 3.66aA	48.10 ± 6.20cB	18.73 ± 3.31aA	1925.25 ± 174.89bB	366.01 ± 27.23aA	7200.19 ± 368.70cB
		Roots	R	14.78 ± 0.74aA	15.90 ± 3.99aA	14.74 ± 4.27aB	1038.03 ± 34.51aB	819.79 ± 50.85bB	3658.46 ± 173.83aA

Conditions: control: Ψ_L_ ≤ 0.5; severe drought stress: Ψ_L_ ≥ 2.0; and 48-h rehydration. RL: Rangpur lime; SM: Sunki Maravilha mandarin; VO: Valencia orange; TAL: Tahiti acid lime. Values are averages ± standard errors (n = 5). Different uppercase letters indicate significant differences between combinations, and different lowercase letters indicate significant differences within the same combination, according to the Scott-Knott test (*p* < 0.05).

**Table 3:** Combinations scions/rootstocks used in the experiment.

Number of combination	Scion	Rootstock	Combinations
1		Rangpur lime	RL (ungrafted)
2		Sunki Maravilha	SM (ungrafted)
3	Sunki Maravilha	Rangpur lime	SM/RL
4	Rangpur lime	Sunki Maravilha	RL/SM
5	Valencia orange	Rangpur lime	VO/RL
6	Valencia orange	Sunki Maravilha	VO/SM
7	Tahiti acid lime	Rangpur lime	TAL/RL
8	Tahiti acid lime	Sunki Maravilha	TAL/SM

RL: Rangpur lime; SM: Sunki Maravilha mandarin; VO: Valencia orange; TAL: Tahiti acid lime.
