# Supplementary material for: Survival strategies of citrus rootstocks subjected to drought
Source: Sci Rep. 2016 Dec 20;6:38775. doi: 10.1038/srep38775 (PMC5171762; doi:10.1038/srep38775)
Supplement: Supplementary Information [file srep38775-s1.doc]

**Supplementary material**

**Survival strategies of citrus rootstocks subjected to drought**

Dayse Drielly Souza Santana-Vieira, Luciano Freschi , Lucas Aragão da Hora Almeida, Diogo Henrique Santos de Moraes, Diana Matos Neves, Liziane Marques dos Santos, Fabiana Zanelato Bertolde, Walter dos Santos Soares Filho, Maurício Antônio Coelho Filho, Abelmon da Silva Gesteira

**
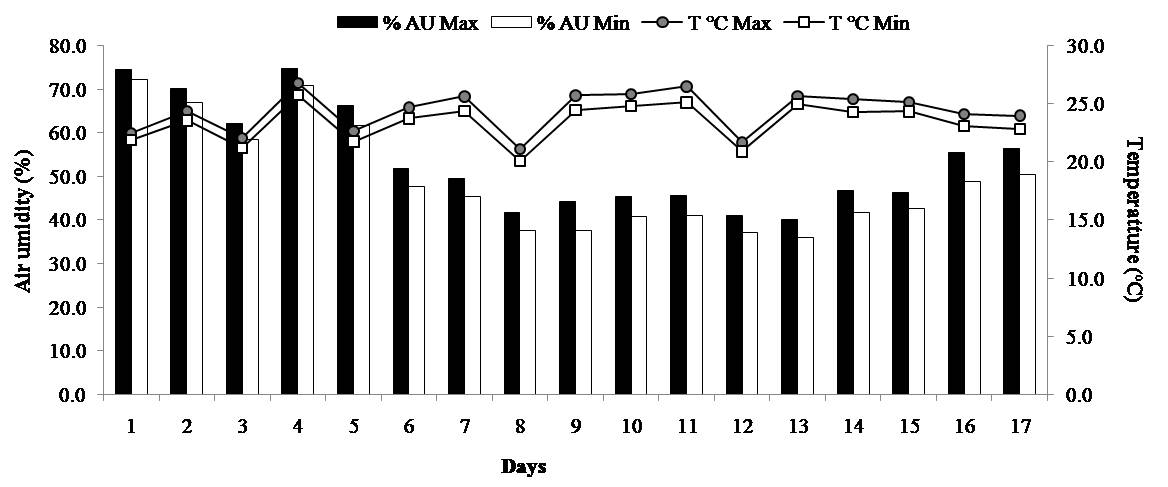
**

**Figure S1** – Microclimate conditions under the screen where the experiment was performed. The daily maximum and minimum temperatures (°C) and air relative humidity (%) for the days of the experiment are represented as lines and bars, respectively.

**
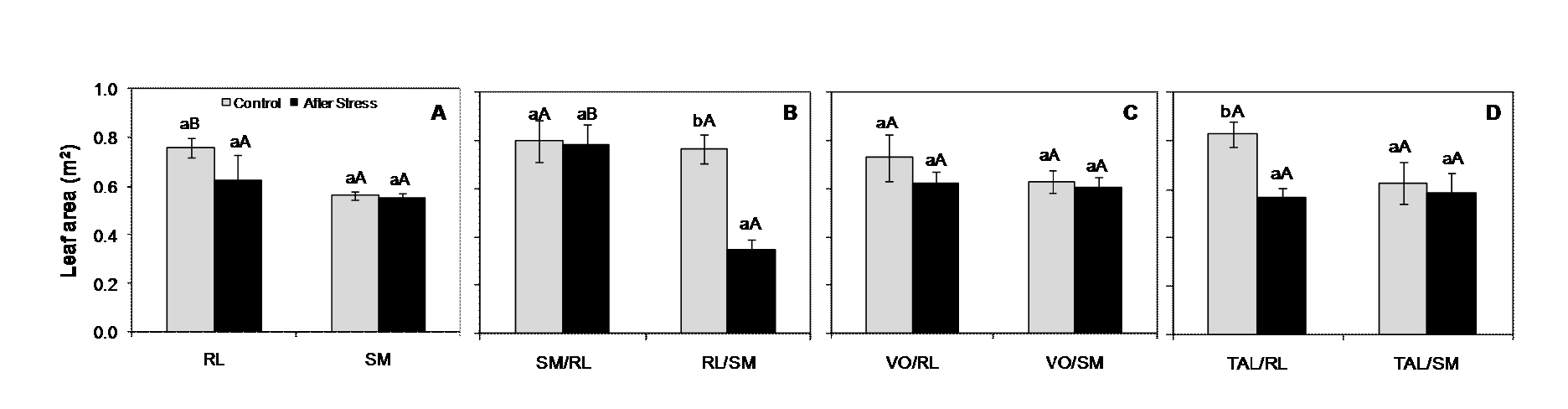
**

**Figure S2** – Plant leaf areas of eight scion/rootstock combinations before (control – gray bars) and after drought stress (after stress – black bars). RL: Rangpur lime; SM: Sunki Maravilha mandarin; VO: Valencia orange; TAL: Tahiti acid lime. Values are averages ± standard errors (n=6). Different uppercase letters indicate significant differences between combinations, and different lowercase letters indicate significant differences within the same combination, according to the Scott-Knott test (*p*<0.05).


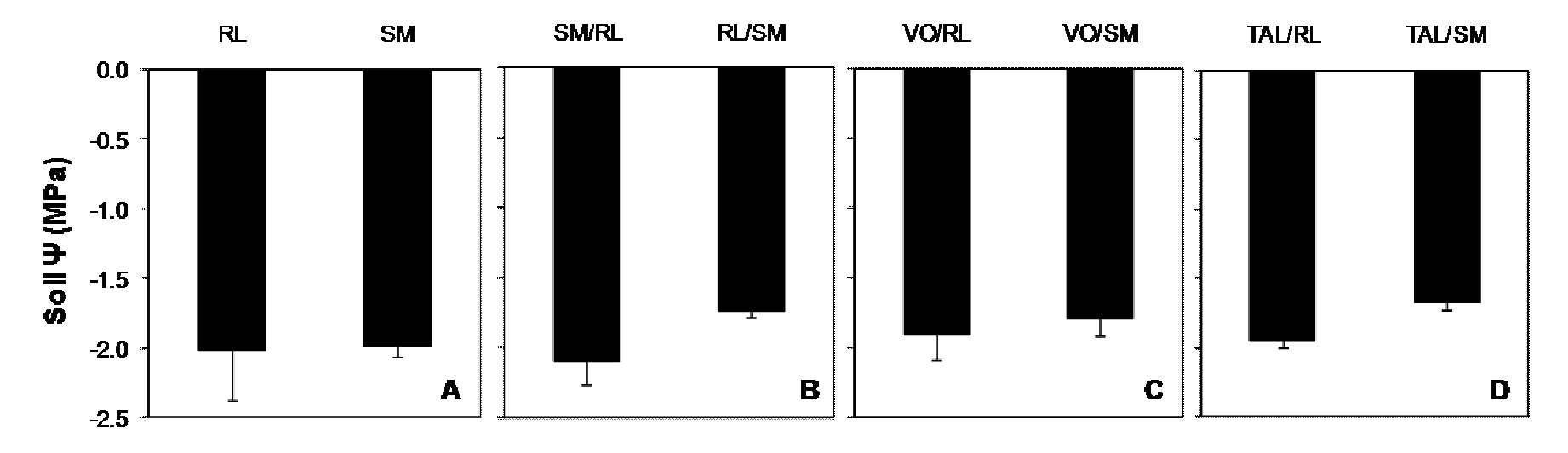


**Figure S3** – Soil matric potential (soil Ψ; MPa) under severe drought stress (Ψ_L_ ≥ 2.0 MPa – black bars) for eight scion/rootstock combinations. RL: Rangpur lime; SM: Sunki Maravilha mandarin; VO: Valencia orange; TAL: Tahiti acid lime. Values are averages (n=3). Error bars indicate standard errors.
